# CDD-RFLNet: Continual Disagreement-Distilled Residual-Feature Learning for Open-World Deepfake-Video Detection

**DOI:** 10.3390/jimaging12100489

**Published:** 2026-10-06

**Authors:** Nomica Choudhry, Imran Rao

**Affiliations:** Faculty of Science, Engineering and Built Environment, Deakin University, Melbourne, VIC 3125, Australia; imranrao@gmail.com

**Keywords:** deepfake-video detection, continual learning, open-set recognition, knowledge distillation, residual-feature learning, teacher disagreement

## Abstract

Deepfake detectors are usually trained on a fixed collection of manipulation methods, although synthetic video generation continues to evolve after deployment. This paper presents CDD-RFLNet, a continual open-world framework that combines RGB, constrained-residual, temporal-change, and forensic-disagreement cues. A calibrated gate uses predictive energy, residual-prototype distance, and temporal disagreement to identify unfamiliar clips. When labeled examples from a new domain become available, a frozen stability teacher preserves earlier knowledge while a plasticity teacher learns the emerging manipulation. Reliability-weighted distillation transfers their shared evidence, and localized disagreement controls lightweight stage adapters. Prototype replay limits forgetting without retaining the full historical training set. Trained initially on FaceForensics++, the model is evaluated through a sequential protocol that introduces unseen manipulation families and datasets. CDD-RFLNet achieves 91.4% unknown-detection AUROC and, after four adaptation stages, 93.7% average AUC with a standard deviation of 0.4% and only 1.0 points of forgetting. These results show that a compact detector can recognize distribution change, learn of emerging attacks, and preserve earlier forensic knowledge.

## 1. Introduction

Deepfake detection is becoming a moving target. New systems for face swapping, facial re-enactment, talking heads, and diffusion-based synthesis continue to appear, producing manipulation traces that differ from those seen during training [1]. The same video may also be compressed, resized, filtered, or processed by a social media platform before it reaches a detector. Such changes can weaken or alter forensic evidence and reduce detection accuracy [2,3]. Consequently, a model that performs well on its original benchmark may fail when the generator, dataset, or acquisition conditions change. Large evaluations conducted in DeepfakeBench and DF40 confirm that this gap between familiar and unseen data remains a central challenge [1,4].

Researchers have addressed this problem by learning evidence that is less tied to a single manipulation method. Face X-Ray models blending boundaries created during face composition [5], while F3-Net and related methods examine frequency information and high-frequency artifacts [6,7]. Other approaches use facial motion [8], spatial and temporal relationships [9,10,11], or synthetic training examples designed to expose common forgery cues [12,13,14,15]. These methods have improved cross dataset generalization, but most still make a closed-set decision: the input is classified as either real or fake.

That binary decision is risky when a detector encounters an unfamiliar attack. An unknown manipulation may be accepted as genuine simply because it does not resemble the fake examples used for training. Recent open-set deepfake research, therefore, adds an explicit unknown category instead of forcing every input into a known class [16]. This is especially important because modern neural networks can produce poorly calibrated probabilities [17], and conventional softmax confidence can remain high for data outside the training distribution [18]. Energy scores and distances in representation space provide useful signals for identifying such unfamiliar inputs [18,19].

Recognizing an unknown manipulation, however, solves only half of the problem. After an analyst verifies and labels examples from a new attack, the detector should learn from them without losing its ability to recognize earlier attacks. Naive fine-tuning can overwrite previously learned knowledge, a problem known as catastrophic forgetting [20,21]. Continual learning reduces this loss through regularization, knowledge distillation, and replaying of representative examples [20,22,23]. Open-set detection and continual learning, therefore, address two connected needs, but they are commonly evaluated as separate tasks. This leaves a practical gap between flagging an unfamiliar video and safely adapting the detector to the new threat.

This paper presents CDD-RFLNet, a framework that connects unknown detection with controlled continual learning. Each video is described through complementary RGB and constrained-residual features, temporal-feature changes, and predicted forensic disagreement. A calibrated gate combines predictive energy [18], distance from known residual prototypes [19], and temporal disagreement to determine whether a clip is familiar. Unfamiliar clips are then selected for limited expert review. When labeled examples become available, a frozen stability teacher preserves the earlier decision structure, while a plasticity teacher learns the emerging manipulation. Reliability-based distillation transfers their shared evidence, and their disagreement determines where compact adapters modify the model. Prototype replay protects earlier knowledge without retaining the complete historical dataset [22,23], while lightweight adapters limit the number of parameters updated during each stage [24,25].

Existing open-set deepfake detectors can identify unfamiliar samples but generally do not provide a mechanism for learning them after deployment [16,26,27]. Conversely, continual deepfake detectors update their representations across predefined tasks but usually assume that the arrival and identity of each new task are already known [28,29,30]. CDD-RFLNet connects these two settings through a shared disagreement signal. During deployment, predicted temporal disagreement contributes to rejecting unfamiliar clips. After verification, disagreement between the stability and plasticity teachers provides a training target and controls where the stage adapters modify the student. The novelty, therefore, lies not in energy scoring, prototype distance, replay, distillation, or low-rank adaptation individually, but in coupling them into a bounded detect–review–adapt–redeploy cycle for open-world deepfake-video detection.

The main contributions are as follows:

- We formulate deepfake-video detection as a complete open-world continual-learning cycle in which unfamiliar manipulations are detected, reviewed, learned, and subsequently recognized under fixed annotation, memory, and parameter budgets.
- We introduce a three-signal open-world gate that combines predictive energy, residual-prototype distance, and temporally concentrated forensic disagreement. The gate is calibrated using only source-domain validation data and does not require target-domain-threshold tuning.
- We develop a disagreement-controlled stability–plasticity distillation mechanism. Teacher reliability determines the classification target, while frame-wise teacher disagreement is retained as supervision and controls the magnitude of stage-specific RGB, residual, and temporal-adapter updates.
- We combine fixed-budget prototype replay with localized low-rank adaptation to preserve earlier forensic-decision geometry without retaining the complete historical-video dataset.
- We evaluate the complete lifecycle through unknown recognition, sequential adaptation, forgetting, forward transfer, calibration, low-false-positive-rate operation, stage-order robustness, memory growth, and deployment efficiency.

## 2. Related Work

### 2.1. Generalizable Video-Deepfake Detection

Deepfake detectors initially focused on spatial artifacts left by a particular generation or compositing process. Face X-Ray learns blending boundaries that can be shared across several face-manipulation methods [5]. F3-Net separates frequency-aware components [6], while high-frequency residual representations reduce the influence of semantic image content [7]. These methods showed that forensic evidence can be found outside ordinary RGB appearance. Their limitation is that fine spatial traces can be weakened by compression, resizing, denoising, and improved synthesis [2,3]. A feature that is highly discriminative on one dataset may, therefore, become unreliable after a change in the generator or media-processing pipeline.

Video methods add temporal evidence that cannot be obtained from independent frames. LipForensics models irregularities in mouth motion [8]; ISTVT uses separate spatial and temporal attention [9]; AltFreezing alternates the optimization of spatial and temporal weights [10]; and TALL arranges successive frames into a compact thumbnail representation [11]. MINTIME addresses changes in face size and the presence of multiple identities [31]. These methods improve video-level reasoning, but their temporal features may still depend on the training manipulations, face-tracking quality, and selected frame interval. They also increase the computational cost of deployment compared with frame-based detectors.

A second line of work improves generalization by broadening the training distribution or learning representations shared by multiple manipulations. Self-Blended Images generates pseudo forgeries from genuine faces without requiring a specific deepfake generator [12]. UCF separates common forensic features from method-specific evidence [13], and LSDA interpolates and extrapolates latent forgery representations [14]. For video, VLB generates blended clips that reproduce facial-feature drift and uses lightweight spatiotemporal adapters to capture both spatial and temporal clues [15]. These approaches improve transfer to manipulations omitted from training, but they still produce a fixed model whose knowledge stops changing after deployment.

Benchmark design has also become an important part of the literature. DeepfakeBench standardizes preprocessing, implementations, datasets, and evaluation protocols [4]. DF40 expands the range of tested manipulations to 40 techniques and shows that conclusions drawn from a small number of older face-swapping datasets do not necessarily transfer to newer synthesis methods [1]. Together, these benchmarks reveal a persistent gap between matched evaluation and performance on unfamiliar data. However, cross-dataset and leave-one-manipulation-out tests measure a single transfer event. They do not show whether a detector can recognize an unknown attack, learn from it, and retain earlier knowledge over a sequence of changes.

### 2.2. Open-Set Recognition and Forensic Uncertainty

Conventional detectors are trained in a closed-set setting and must classify every input as real or fake. This assumption is unsafe when a manipulation is unlike the training data because softmax confidence can remain high for an out-of-distribution input. Temperature scaling improves the calibration of known class probabilities [17], but calibration alone does not guarantee recognition of unknown data. Energy-based scoring uses the classifier logits to separate familiar and unfamiliar inputs [18], while Mahalanobis distance measures how far a test feature lies from known class distributions [19]. The two signals capture different information, although both remain sensitive to the quality and coverage of the validation distribution used to set their thresholds.

Open-set ideas have recently entered multimedia forensics. Bahavan et al. use supervised contrastive learning to separate genuine media, known deepfakes, and deepfakes produced by unknown manipulation methods [16]. SeeABLE instead treats deepfake exposure as a one-class out-of-distribution problem [32]. Forensic Self-Descriptions models residual microstructures and supports zero-shot detection, open-set source attribution, and clustering without training on the tested generators [26]. Face-Trace combines energy-based rejection with progressive clustering of rejected synthetic face images, so previously unseen generators can be discovered as samples arrive [27].

These studies establish that an explicit unknown outcome is preferable to a forced binary decision. Nevertheless, most operate on images or source attribution rather than video-level real or fake detection. Also, rejection and clustering do not by themselves update the detector that produced the unknown decision. Moreover, uncertainty does not transfer reliably across domains: recent experiments report that the relationship between uncertainty and prediction error can weaken or even invert on unseen deepfake datasets [33]. A deployable gate, therefore, needs complementary evidence and must be evaluated together with the adaptation process that follows a rejection.

### 2.3. Continual Learning and Evolving Forensics

Continual learning studies how a model can acquire new knowledge without catastrophically forgetting earlier tasks. Regularization methods such as Elastic Weight Consolidation limit changes to parameters considered important for previous tasks [20]. Distillation methods such as Learning without Forgetting preserve the predictions of an earlier model while learning new classes [21]. Replay methods retain a small part of the past: iCaRL stores representative exemplars and class prototypes [22], whereas Dark Experience Replay also preserves earlier logits [23]. These strategies make different compromises. Regularization and distillation avoid a large memory but may accumulate errors; replay is generally more stable but introduces storage, privacy, and sample-selection concerns. Parameter-efficient adapters and low-rank updates offer a further option by restricting how much of the network can change [24,25].

Several studies have adapted these ideas to synthetic-media detection. CoReD uses teacher/student distillation for sequential adaptation across deepfake and GAN generated face domains [28]. CDDB provides easy, hard, and long task sequences for measuring continual deepfake detection and forgetting [29].

Building on this benchmark, Prompt2Guard uses conditioned prompts with a frozen-vision language model to achieve exemplar-free continual detection on CDDB [30]. Chronological evaluation over seven years of generators further shows that continual updating can preserve earlier accuracy, but performance on a future generator before adaptation remains close to random [34]. Face-D^2^CL combines spatial, wavelet, and Fourier evidence with two replay-free constraints that balance adaptation and retention [35]. Continually refreshed adversarial training has also been evaluated across large collections of public image and video datasets [36].

This literature confirms that continual adaptation is feasible, but most protocols assume that the next task has already been identified and its samples have already been labeled. They do not decide which incoming videos are unknown, which should be sent for review, or when an update should begin. In addition, much of the continual deepfake literature concerns generated images or frame-based classification. Temporal evidence, calibrated low-false-positive-rate operation, annotation budgets, and the deployment cost of repeated video updates remain less developed.

### 2.4. Synthesis and Research Gap

The literature leaves three parts of the deployment problem insufficiently connected. Generalizable detectors improve transfer but remain fixed. Open-set methods reject or cluster unfamiliar samples but usually stop before supervised adaptation. Continual methods learn new domains but generally assume that task boundaries and labels are already available. The closest progressive systems either discover unknown image generators without adapting a video detector [27], or continually refresh a detector without an explicit sample-level gate and controlled-forgetting mechanism [36].

CDD-RFLNet addresses this gap as one sequential forensic process. A video is first assessed using predictive energy, residual-prototype distance, and temporal disagreement. Rejected clips enter a limited-review budget rather than being treated as genuine. After verification, stability and plasticity teachers separate evidence that should be preserved from evidence that should change, and their disagreement controls compact adapters. Prototype replay limits forgetting under a fixed memory. The resulting evaluation joins unknown recognition, continual adaptation, forgetting, forward transfer, calibration, low-false-positive-rate operation, and deployment growth within the same sequence of manipulation families.

## 3. Problem Formulation

Let a video be decomposed into face clips

(1) $$X={\left\{x_{t}\right\}}_{t=1}^{T},\;x_{t}\in {\left[0,1\right]}^{3\times H\times W},$$

with binary authenticity label y∈{0,1} and manipulation-family label $c\in \{0,1,\ldots ,C_{s}\}$. Class c=0 denotes genuine media; the remaining indices denote fake families known after stage *s*. At stage s+1, the stream may contain an unknown family $c\notin \{0,\ldots ,C_{s}\}$.

The learner receives a sequence

(2) $$S=\left(D_{0},D_{1},\ldots ,D_{S}\right),$$

where $D_{0}$ is the source-training domain and each later stage contains a small labeled set from one or more emerging manipulation families. Before labels are available, the deployed detector must decide whether an incoming clip is familiar. After a bounded set of rejected clips is annotated, the model is updated without direct access to the complete previous training data.

After stage *s*, the detector returns three outputs:

(3) $$f_{s}\left(X\right)=\left(\hat{y},\hat{c},u\left(X\right)\right),$$

where $\hat{y}$ is the real/fake probability, $\hat{c}$ is the known-family prediction, and u(X) is the unknown score. The learning objective must jointly maximize current-stage accuracy, retain earlier accuracy, and maintain reliable unknown rejection under a fixed memory and parameter budget.

## 4. Proposed Method

### 4.1. Architecture Overview

**Figure 1:**
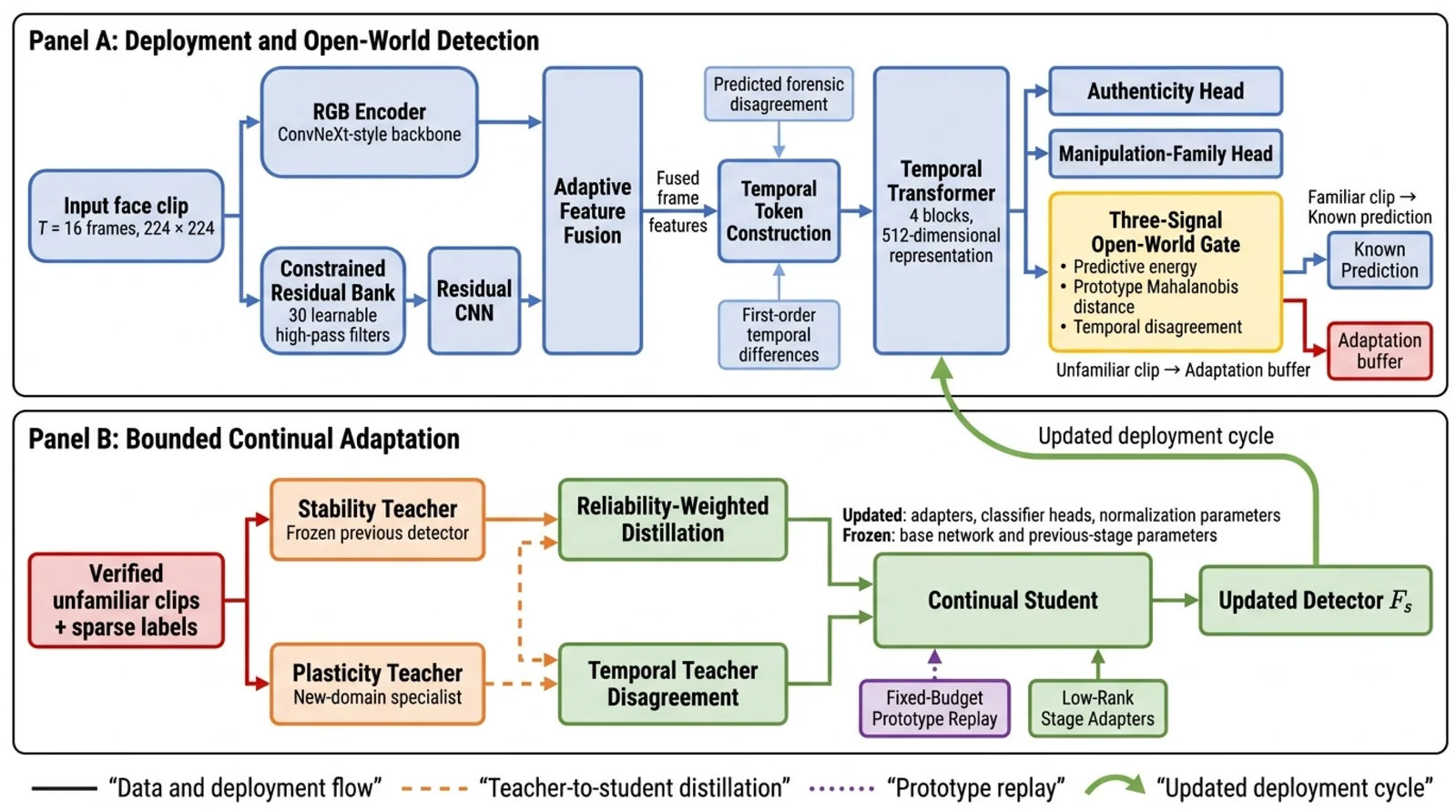
Overall architecture of CDD-RFLNet. Panel (**A**) shows deployment and open-world detection, while Panel (**B**) presents bounded continual adaptation.

The proposed Continual Disagreement-Distilled Residual-Feature Learning Network (CDD-RFLNet) formulates deepfake detection as a sequential open-world learning problem rather than a fixed binary classification task. Figure 1 illustrates its two connected phases: deployment and bounded continual adaptation. During deployment, the framework classifies an incoming face clip when it matches the learned manipulation domains. An unfamiliar clip is instead marked as unknown and transferred to an adaptation buffer for review. During adaptation, a limited set of verified clips from this buffer is used to learn the emerging manipulation while preserving previously acquired forensic knowledge.

At the source stage (s=0), the detector processes T=16 aligned RGB frames of size 224×224. Each frame is analyzed by two complementary convolutional branches. A ConvNeXt-style RGB encoder learns semantic, texture, and appearance cues, whereas a constrained-residual bank followed by a shallow-residual CNN emphasizes local prediction errors and high-frequency manipulation traces. A learned gate fuses the two feature streams on a frame-by-frame basis. The fused features, their first-order temporal changes, and a predicted forensic disagreement value are then supplied to a four-block temporal Transformer with a 512-dimensional embedding. The Transformer produces a video representation that supports binary authenticity prediction and known manipulation-family classification.

The deployed model is also equipped with a three-signal open-world gate. The gate combines predictive energy, distance from known residual-feature prototypes, and temporal forensic disagreement. This combination prevents an unfamiliar manipulation from being forced into either the genuine class or a previously learned fake class. Once enough rejected clips have been verified, the current checkpoint is copied into a frozen *stability teacher*, while a second copy is adapted to the new domain to form a *plasticity teacher*. Their reliability-weighted predictions supervise a continual student, and their localized disagreement controls lightweight low-rank adapters. Both teachers are discarded after the update; therefore, only the compact student is retained at inference.

The architecture is not a simple sequential combination of established components. Its central coupling variable is forensic disagreement. At deployment, the predicted disagreement ${\hat{d}}_{t}$ contributes to the unknown score used to route unfamiliar clips for review. During adaptation, the measured teacher disagreement $d_{t}^{⋆}$ supervises the disagreement head and controls the strength of the stage-specific adapter update. Consequently, frames that remain compatible with earlier forensic knowledge receive limited modification, whereas frames exhibiting strong old–new conflict receive greater adaptation. This coupling connects open-world recognition directly with localized continual learning.

### 4.2. Dual-Domain Frame Representation

Let $X={\left\{x_{t}\right\}}_{t=1}^{T}$ denote an aligned face clip. Each frame is processed by an RGB encoder $E_{S}$ and a residual encoder $E_{R}$:

(4) $$s_{t}=E_{S}\left(x_{t}\right),\;r_{t}=E_{R}\;\left(H_{\phi }\left(x_{t}\right)\right).$$

**Figure 2:**
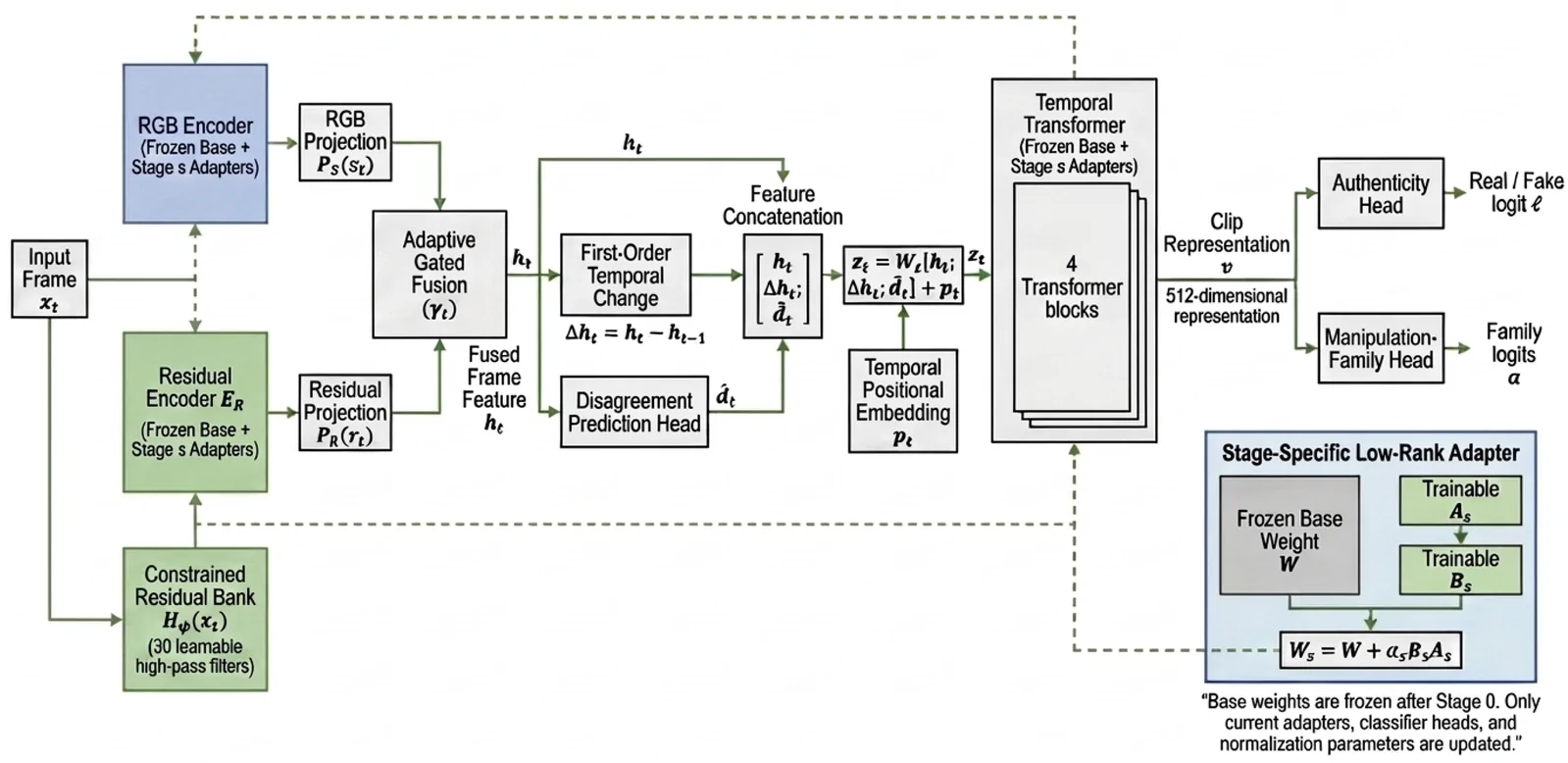
Internal deep learning architecture of CDD-RFLNet. The RGB and constrained-residual branches extract complementary spatial evidence, which is combined through adaptive gated fusion. Fused features, temporal changes, and predicted disagreement are integrated by the temporal Transformer. Stage-specific low-rank adapters support parameter-efficient continual adaptation while preserving the frozen base model.

The spatial encoder $E_{S}$ follows a compact ConvNeXt-style design. Its depthwise spatial convolutions capture local facial patterns, while layer normalization, pointwise channel projections, GELU activations, and residual connections progressively learn texture and appearance representations. This branch is particularly responsive to visible artifacts such as unnatural skin texture, blending, and inconsistent facial details. Figure 2 presents the internal deep learning architecture of CDD-RFLNet and the placement of its stage-specific low-rank adapters.

The forensic branch first applies a bank $H_{\phi }={\left\{H^{\left(k\right)}\right\}}_{k=1}^{K}$ of K=30 constrained 5×5 prediction filters. For every kernel, the center coefficient and the remaining coefficients satisfy

(5) $$H_{0,0}^{\left(k\right)}=-1,\;\sum_{\left(i,j)\neq (0,0\right)}H_{i,j}^{\left(k\right)}=1.$$

Consequently, a filter response approximates the difference between the center pixel and a learned prediction obtained from its neighborhood. Slowly varying semantic content is suppressed, whereas resampling artifacts, blending boundaries, abnormal noise, and other high-frequency traces are enhanced. The responses of the 30 filters are stacked and passed through the shallow-residual CNN $E_{R}$, whose skip-connected convolutional blocks preserve weak forensic signals while producing the residual descriptor $r_{t}$.

Because the two branches have different feature statistics, their outputs are first mapped to a common latent dimension by learned projections $P_{S}$ and $P_{R}$. A frame-dependent fusion gate then determines their relative contributions:

(6) $$\begin{array}{cc} \gamma_{t} & =\sigma \;\left(W_{g}\left[s_{t};r_{t}\right]+b_{g}\right), \end{array}$$

(7) $$\begin{array}{cc} h_{t} & =\gamma_{t}⊙P_{S}\left(s_{t}\right)+\left(1-\gamma_{t}\right)⊙P_{R}\left(r_{t}\right), \end{array}$$

where [;] denotes concatenation and ⊙ denotes element-wise multiplication. Unlike fixed concatenation or averaging, $\gamma_{t}$ allows the detector to rely more strongly on semantic evidence when visual artifacts are clear and on residual evidence when the manipulation is perceptually subtle.

At the source stage, auxiliary heads attached to the two branches produce frame-level authenticity probabilities $p_{t}^{S}$ and $p_{t}^{R}$. Their absolute difference provides a self-supervised source disagreement target:

(8) $$d_{t}^{\left(0\right)}=\left|p_{t}^{S}-p_{t}^{R}\right|.$$

A lightweight disagreement predictor $G_{d}$, operating before temporal aggregation, estimates

(9) $${\hat{d}}_{t}=\sigma \;\left(G_{d}\left([P_{S}\left(s_{t}\right);P_{R}\left(r_{t}\right)]\right)\right).$$

This causal definition is important because ${\hat{d}}_{t}$ is subsequently used as an input to the temporal model. At s=0, $G_{d}$ is supervised by $d_{t}^{\left(0\right)}$; at later stages, it is supervised by the disagreement between the stability and plasticity teachers, as defined in Section 4.6.

### 4.3. Temporal-Change and Video-Level Modeling

Independent frame predictions may overlook brief or temporally inconsistent manipulations. CDD-RFLNet, therefore, augments the fused representation with its first-order temporal change:

(10) $$\Delta h_{t}=\left\{\begin{array}{cc} 0, & t=1,\\ h_{t}-h_{t-1}, & t>1. \end{array}\right.$$

For each frame, the fused appearance feature, temporal difference, and predicted disagreement are combined into a temporal token:

(11) $$z_{t}=W_{z}\left[h_{t};\Delta h_{t};{\hat{d}}_{t}\right]+p_{t},$$

where $W_{z}$ projects the concatenated vector to d=512 and $p_{t}$ is a learned positional embedding.

The sequence ${\left\{z_{t}\right\}}_{t=1}^{T}$ is processed by L=4 Transformer encoder blocks. Each block contains pre-normalized multi-head self-attention and a two-layer feed-forward network with residual connections:

(12) $$\begin{array}{cc} q_{t}^{\left(\ell \right)} & =u_{t}^{\left(\ell -1\right)}+\mathrm{MHSA}\;\left(\mathrm{LN}(u_{t}^{\left(\ell -1\right)})\right), \end{array}$$

(13) $$\begin{array}{cc} u_{t}^{\left(\ell \right)} & =q_{t}^{\left(\ell \right)}+\mathrm{MLP}\;\left(\mathrm{LN}(q_{t}^{\left(\ell \right)})\right),\;\ell =1,\ldots ,L, \end{array}$$

with $u_{t}^{\left(0\right)}=z_{t}$. Self-attention permits evidence from any sampled frame to be compared with evidence from every other frame, which is useful for detecting inconsistent motion, transient synthesis errors, and manipulation traces that appear only in a short segment. The output tokens are aggregated into a video-level representation

(14) $$v=\mathrm{Pool}\left(u_{1}^{\left(L\right)},\ldots ,u_{T}^{\left(L\right)}\right).$$

An authenticity head produces the binary prediction $\hat{y}=\sigma \left(w_{y}^{⊤}v+b_{y}\right)$, while a family head returns logits $a\in R^{C_{s}+1}$ for the genuine class and the $C_{s}$ manipulation families known after stage *s*. The probabilities are temperature-calibrated on validation data before being used for confidence estimation and open-world screening.

### 4.4. Disagreement-Controlled Stage Adapters

Updating the entire network for every emerging manipulation would increase storage and make catastrophic forgetting more likely. After Stage 0, CDD-RFLNet, therefore, inserts LoRA-style low-rank adapters [25] into selected projections of the RGB encoder, residual encoder, and temporal Transformer. For a base projection $W^{\left(0\right)}$, the effective weight after stage *s* is

(15) $$W^{\left(s\right)}=W^{\left(0\right)}+\sum_{i=1}^{s}\alpha_{i}B_{i}A_{i},\;A_{i}\in R^{r_{i}\times d_{\mathrm{in}}},\;B_{i}\in R^{d_{\mathrm{out}}\times r_{i}},$$

where $r_{i}\ll \min\left(d_{\mathrm{in}},d_{\mathrm{out}}\right)$. We use rank $r_{i}=8$ for the RGB and temporal projections and rank $r_{i}=4$ for the residual branch. The quantity $\alpha_{i}$ is a fixed, rank-normalized LoRA scaling coefficient rather than a gradient-updated parameter:

(16) $$\alpha_{i}=\frac{\alpha_{\mathrm{LoRA}}}{r_{i}},\;\alpha_{\mathrm{LoRA}}=16.$$

Consequently, $\alpha_{i}=2$ for the rank-8 RGB and temporal adapters and $\alpha_{i}=4$ for the rank-4 residual adapter. Only the low-rank matrices $A_{i}$ and $B_{i}$ are optimized by gradient descent. When stage *s* is learned, $W^{\left(0\right)}$ and all adapters from earlier stages remain frozen; only $\left(A_{s},B_{s}\right)$, selected normalization parameters, and the output heads are optimized.

The new adapter is activated according to the predicted frame-level forensic disagreement:

(17) $$g_{t}=\sigma \left(\beta_{0}+\beta_{1}{\hat{d}}_{t}\right),\;{\tilde{h}}_{t}=h_{t}+g_{t}\;A_{s}\left(h_{t}\right),$$

where $A_{s}$ denotes the aggregate transformation introduced at the current stage. The quantities $\beta_{0}$ and $\beta_{1}$ are fixed scalar hyperparameters, not learnable parameters. We use $\beta_{0}=-2$ and $\beta_{1}=4$, selected on the held-out Stage 1 validation subset and then frozen for all subsequent stages. Thus, the adapter activation increases monotonically from σ(−2)≈0.119 when ${\hat{d}}_{t}=0$ to σ(2)≈0.881 when ${\hat{d}}_{t}=1$. Frames on which the old and new forensic evidence agree, therefore, receive only a small residual update, whereas frames with high disagreement activate the adapter more strongly. This localizes plasticity instead of modifying the complete representation of every frame.

### 4.5. Three-Signal Open-World Gate

The open-world gate combines predictive, distributional, and temporal evidence. The first component is the energy of the genuine and known manipulation-family logits [18]:

(18) $$u_{\mathrm{eng}}\left(X\right)=-\tau_{e}\log\sum_{j=0}^{C_{s}}\exp\;\left(a_{j}/\tau_{e}\right),$$

where larger energy indicates weaker support from the known classes. The quantity $\tau_{e}>0$ is the energy temperature controlling the smoothness of the log-sum-exp aggregation. Smaller values make the score more strongly determined by the largest class logit, whereas larger values distribute the contribution more evenly across the known classes. We use $\tau_{e}=1.0$, which is selected using only the FF++ source-validation calibration episodes and subsequently frozen for all target datasets and continual-learning stages.

The second component measures the distance between the video representation and the nearest known-class prototype. For every class *j*, the framework stores a mean $\mu_{j}$ and a regularized diagonal covariance $\Sigma_{j}$. The prototype score is the minimum Mahalanobis distance:

(19) $$u_{\mathrm{pro}}\left(X\right)=\min_{0\leq j\leq C_{s}}{\left(v-\mu_{j}\right)}^{⊤}\Sigma_{j}^{-1}\left(v-\mu_{j}\right).$$

The third component summarizes both persistent and temporally localized disagreement:

(20) $$u_{\mathrm{dis}}\left(X\right)=\frac{1}{T}\sum_{t=1}^{T}{\hat{d}}_{t}+\kappa \max_{1\leq t\leq T}{\hat{d}}_{t}.$$

Here, κ≥0 is a fixed scalar that controls the contribution of the largest frame-level disagreement relative to the clip-average disagreement. We use κ=0.5, selected using the source-validation calibration episodes and then frozen. The mean term captures disagreement sustained throughout the clip, while the maximum term preserves sensitivity to a short unfamiliar segment that could otherwise be hidden through temporal averaging. Because ${\hat{d}}_{t}\in \left[0,1\right]$, the contribution of a single frame is bounded by κ, limiting sensitivity to isolated noisy frames.

We examined $\tau_{e}\in \left\{0.5,1.0,2.0\right\}$ and κ∈{0,0.25,0.5,0.75,1.0} using only the FF++ source-validation calibration episodes. The selected values, $\tau_{e}=1.0$ and κ=0.5, provided the best validation trade-off between unknown-detection AUROC and FPR@95TPR. The three-signal gate retained its advantage over the energy-plus-prototype configuration throughout the examined ranges. In particular, κ=0 reduced sensitivity to temporally localized manipulations, whereas larger values placed excessive weight on isolated high-disagreement frames. No target-domain test samples were used in this sensitivity analysis or in hyperparameter selection.

To place the three quantities on comparable scales, each score is robustly standardized using the median $m_{k}$ and interquartile range $q_{k}$ estimated from the Stage-0 validation set:

(21) $${\bar{u}}_{k}\left(X\right)=\frac{u_{k}\left(X\right)-m_{k}}{q_{k}+\epsilon },\;k\in \left\{\mathrm{eng},\mathrm{pro},\mathrm{dis}\right\}.$$

The final unknown score is

(22) $$u\left(X\right)=w_{e}{\bar{u}}_{\mathrm{eng}}\left(X\right)+w_{p}{\bar{u}}_{\mathrm{pro}}\left(X\right)+w_{d}{\bar{u}}_{\mathrm{dis}}\left(X\right),\;w_{e},w_{p},w_{d}\geq 0,\;w_{e}+w_{p}+w_{d}=1.$$

#### Training and Freezing of the Open-World Gate

The open-world gate was calibrated once, after completing Stage 0 and before evaluating or adapting to any target dataset. Only the official FF++ validation split was used for this calibration. We constructed four leave-one-manipulation-family-out episodes. In each episode, one of the four FF++ manipulation families was treated as pseudo-unknown, whereas genuine videos and the remaining three manipulation families were treated as known. Consequently, no examples from Celeb-DF-v2, DFDC, DeeperForensics, WildDeepfake, or DF40-FS were used to train or tune the gate.

For every episode, the energy, prototype-distance, and temporal-disagreement scores were standardized using the medians and interquartile ranges calculated from its known validation clips. Let $K_{r}$ and $U_{r}$ denote the known and pseudo-unknown clips, respectively, in episode *r*. The non-negative fusion weights $w=(w_{e},w_{p},w_{d})$ were parameterized as w=softmax(η) and estimated over the four pooled calibration episodes using the pairwise logistic-ranking objective

(23) $$L_{\mathrm{gate}}=\frac{1}{4}\sum_{r=1}^{4}\frac{1}{|K_{r}\left|\right|U_{r}|}\sum_{X_{k}\in K_{r}}\sum_{X_{u}\in U_{r}}\log\;\left[1+\exp\;\left(-\left(u\left(X_{u}\right)-u\left(X_{k}\right)\right)\right)\right].$$

This objective encourages pseudo-unknown clips to receive larger open-world scores than known clips while preserving the simplex constraint in Equation (22).

The gate parameters were initialized with η=(0,0,0), corresponding to equal initial fusion weights, and optimized using Adam for 500 iterations with a learning rate of $1\times 10^{-2}$, $\beta_{1}=0.9$, $\beta_{2}=0.999$, and no weight decay. Early stopping with a patience of 30 iterations was applied according to the pooled leave-one-manipulation-family-out validation loss. The resulting seed-averaged fusion weights were $w_{e}=0.30$, $w_{p}=0.35$, and $w_{d}=0.35$.

After estimating the fusion weights, the decision threshold was defined as the 95th percentile of the open-world scores of known FF++ validation clips,

(24) $$\delta =Q_{0.95}\left(\left\{u\left(X\right):X\in K_{\mathrm{val}}\right\}\right),$$

which corresponds to a target false-rejection rate of 5% on known source clips. The fusion weights, robust-normalization statistics, and threshold were then frozen and were not re-estimated on any target test domain.

When a new manipulation family was subsequently verified and introduced during continual learning, only its labeled training examples were used to construct its class-prototype statistics. Target test clips were never used for gate calibration or prototype estimation. The gate-fusion weights and the original Stage 0 normalization parameters remained unchanged throughout all continual-learning stages.

The frozen three-signal gate achieved an unknown-detection AUROC of 91.4%, an AUPR of 90.2%, an FPR@95 of 22.8%, and an OSCR of 83.6% on unseen manipulation families and datasets. Relative to the energy-plus-prototype configuration, incorporating temporal disagreement increased AUROC by 4.7 percentage points and reduced FPR@95 by 11.8 percentage points, demonstrating that the learned source-domain fusion generalized to previously unseen forged videos.

### 4.6. Reliability-Weighted Stability–Plasticity Distillation

At stage s>0, the previously deployed detector is copied and frozen as the stability teacher, $F^{\mathrm{stb}}=F_{s-1}$. A second copy is optimized on the new labeled set $D_{s}$, using class-balanced spatial, compression, and temporal augmentations, to form the plasticity teacher $F^{\mathrm{pla}}$. Both teachers, therefore, use the same dual-domain Transformer backbone as the student, but they represent different objectives: $F^{\mathrm{stb}}$ preserves the old decision structure, whereas $F^{\mathrm{pla}}$ specializes in the emerging manipulation.

Let $p_{t}^{\mathrm{stb}}$ and $p_{t}^{\mathrm{pla}}$ denote their calibrated frame-level authenticity probabilities. For teacher b∈{stb,pla}, reliability is obtained from predictive entropy:

(25) $$\begin{array}{cc} H(p) & =-p\log(p+\epsilon )-(1-p)\log(1-p+\epsilon ), \end{array}$$

(26) $$\begin{array}{cc} \rho_{t}^{\left(b\right)} & =\exp\;\left[-H\left(p_{t}^{\left(b\right)}\right)/\tau_{u}\right]. \end{array}$$

Here, $\tau_{u}>0$ is the reliability temperature controlling how sharply predictive entropy is converted into teacher confidence, and ϵ is a small constant used for numerical stability. A confident teacher, consequently, contributes more strongly to the soft target:

(27) $$q_{t}=\frac{\rho_{t}^{\mathrm{stb}}p_{t}^{\mathrm{stb}}+\rho_{t}^{\mathrm{pla}}p_{t}^{\mathrm{pla}}}{\rho_{t}^{\mathrm{stb}}+\rho_{t}^{\mathrm{pla}}+\epsilon }.$$

Teacher conflict is retained separately rather than being removed by averaging:

(28) $$d_{t}^{★}=\left|p_{t}^{\mathrm{stb}}-p_{t}^{\mathrm{pla}}\right|.$$

The student learns $q_{t}$ as its classification target and $d_{t}^{⋆}$ as the target of the disagreement predictor in Equation (9). Thus, reliability determines *which teacher should be trusted*, while disagreement determines *where the student should change*. The teachers are required only during training and impose no additional inference cost.

### 4.7. Fixed-Budget Prototype Replay and Continual Objective

CDD-RFLNet retains compressed feature tokens rather than decoded historical videos. Let *B* denote the fixed total-replay capacity. After stage *s*, the budget allocated to each genuine or known manipulation class is

(29) $$M_{s}=\left\lfloor \frac{B}{C_{s}+1}\right\rfloor .$$

Farthest-point sampling selects diverse tokens at the input to the adaptable projection. For each retained token, the memory stores its class label and stability-teacher logit; the memory additionally maintains class means and diagonal covariances. Whenever a new class is introduced, all classes are resampled under the same total budget so that memory usage does not grow with the number of stages.

The continual student is optimized using

(30) $$L_{\mathrm{cont}}=L_{\mathrm{new}}+\lambda_{\mathrm{kd}}L_{\mathrm{KD}}+\lambda_{\mathrm{dis}}L_{\mathrm{dis}}+\lambda_{\mathrm{rel}}L_{\mathrm{rel}}+\lambda_{\mathrm{rep}}L_{\mathrm{rep}}+\lambda_{\mathrm{unk}}L_{\mathrm{unk}}+\lambda_{\mathrm{sp}}L_{\mathrm{sp}}.$$

Here, $L_{\mathrm{new}}$ combines binary authenticity and manipulation-family cross-entropy losses on $D_{s}$. The teacher-guided terms are

(31) $$\begin{array}{cc} L_{\mathrm{KD}} & =\frac{1}{T}\sum_{t=1}^{T}\mathrm{KL}\left(\mathrm{Bern}\left(q_{t}\right)∥\mathrm{Bern}\left(p_{t}^{S}\right)\right), \end{array}$$

(32) $$\begin{array}{cc} L_{\mathrm{dis}} & =\frac{1}{T}\sum_{t=1}^{T}\left|{\hat{d}}_{t}-d_{t}^{⋆}\right|, \end{array}$$

where $p_{t}^{S}$ is the student probability. $L_{\mathrm{rel}}$ preserves the pairwise arrangement of earlier class prototypes. If $D^{\mathrm{stb}}$ and $D^{S}$ are the normalized pairwise prototype-distance matrices of the stability teacher and student, respectively, then

(33) $$L_{\mathrm{rel}}={∥D^{S}-D^{\mathrm{stb}}∥}_{F}^{2}.$$

$L_{\mathrm{rep}}$ applies authenticity and family classification to the stored replay tokens. To regularize the open-world boundary, known replay features $X_{K}$ are constrained below margin $m_{K}$, whereas hard open-space features ${\tilde{X}}_{U}$, obtained through prototype interpolation and covariance-based extrapolation, are constrained above $m_{U}>m_{K}$:

(34) $$L_{\mathrm{unk}}={\left[u\left(X_{K}\right)-m_{K}\right]}_{+}+{\left[m_{U}-u\left({\tilde{X}}_{U}\right)\right]}_{+},$$

where ${\left[z\right]}_{+}=\max\left(0,z\right)$. Finally, $L_{\mathrm{sp}}$ imposes group sparsity on new adapter activations so that only necessary channels are changed.

### 4.8. Stage-Wise Training and Inference

Training and deployment proceed as follows.

1. **Source learning:** Train $F_{0}$ on the source domain. Supervise the disagreement predictor using Equation (8), and estimate the class prototypes, robust score statistics, gate weights, and threshold using only the source training and validation partitions.
2. **Deployment and rejection:** Apply $F_{s-1}$ to each incoming clip. If u(X)≤δ, return its calibrated familiar-class decision. Otherwise, assign the clip an unknown status and retain only its compressed representation in the review buffer. Cluster the buffer so that near-duplicate clips do not cause repeated annotation effort.
3. **Bounded verification:** Select a fixed number of cluster representatives for expert review. Begin an update only when an emerging manipulation family has accumulated enough verified examples.
4. **Controlled adaptation:** Freeze $F_{s-1}$ as the stability teacher, train the plasticity teacher on $D_{s}$, initialize the stage-*s* adapters, and optimize Equation (30) using the new labeled clips together with the fixed-replay memory. The base encoders and all earlier adapters remain frozen.
5. **Redeployment:** Update the verified-family prototypes, rebalance the fixed-replay memory, discard both teachers, and deploy the resulting student as $F_{s}$.

At inference, the deployed system contains only the dual-domain encoders, fusion gate, disagreement predictor, accumulated adapter bank, temporal Transformer, classification heads, open-world gate, and current prototype statistics. For a long video, uniformly sampled clips are evaluated independently. Their authenticity logits are averaged to obtain the video-level detection result, whereas the maximum clip-level unknown score is retained so that a short unfamiliar manipulation cannot be obscured by a larger number of familiar frames.

## 5. Experimental Protocol

### 5.1. Datasets and Continual Sequence

Stage 0 uses FaceForensics++ (FF++) c23 [2], including genuine videos and the DeepFakes, Face2Face, FaceSwap, and NeuralTextures manipulation families. We follow the official subject-disjoint FF++ split, comprising 3600 training videos, 700 validation videos, and 700 test videos across the genuine class and four manipulation classes.

The primary continual-learning sequence is

(35) FF++→Celeb-DF-v2→DFDC→DeeperForensics→DF40-FS.

Table 1 summarizes the video allocation used at each stage. At every target stage, the learner receives K∈{10,25,50,100} labeled videos from the emerging manipulation family. A separate set of 100 videos is used for validation, while 500 videos are retained for held-out evaluation. The labeled adaptation videos are removed from the evaluation pool. Identity and original-video groups remain disjoint across training, validation, replay-memory construction, and testing.

WildDeepfake (WDF) [37] is used only for the Stage-0 zero-shot evaluation and is not used for training, validation, gate calibration, or continual adaptation.

The replay memory has a fixed total capacity of B=100 embeddings. At Stage 0, the genuine class and four FF++ manipulation classes each receive embeddings. As new manipulation classes are introduced, the per-class allocation decreases according to Equation (29), while the total replay capacity remains fixed. Three independent seeds use the same primary-stage order, and five additional random orders are used to assess order sensitivity.


(36) $$M_{0}=\left\lfloor \frac{100}{5}\right\rfloor =20$$

### 5.2. Implementation Details

All methods use the same video decoding, face preprocessing, and clip-sampling pipeline. We uniformly sample T=16 frames from each video, align the detected face region, resize each crop to 224×224 pixels, and apply the normalization used by the ImageNet-pretrained RGB backbone. During training, the same class-balanced spatial, compression, and temporal augmentations are applied to the proposed method and all retrained baselines. No test-time augmentation is used. Validation and test clips are processed deterministically using uniform frame sampling and the same alignment, resizing, and normalization operations.

The Stage-0 detector uses an RGB ConvNeXt-style encoder, 30 constrained 5×5 residual filters, a shallow-residual encoder, and a four-block temporal Transformer with an embedding dimension of 512. The disagreement head predicts one scalar per frame. Stage adapters use rank r=8 for the RGB and temporal projections and rank r=4 for the residual branch.

The plasticity teacher is trained for 10 epochs. The continual student and all retrained continual-learning baselines are trained for 15 epochs using AdamW, an initial learning rate of $5\times 10^{-5}$, weight decay of 0.05, and a mini-batch size of eight video clips. We use a five-epoch linear warm-up followed by cosine learning-rate decay and class-balanced mini-batches. Unless an ablation explicitly varies the memory size, the replay budget is fixed at B=100 embeddings. The proposed loss weights are

(37) $$\left(\lambda_{kd},\lambda_{dis},\lambda_{rel},\lambda_{rep},\lambda_{unk},\lambda_{sp}\right)=\left(1,0.5,1,0.5,0.5,10^{-4}\right).$$

Table 2 summarizes the implementation settings.

The continual-learning loss weights and optimization settings were selected using the FF++ source-validation split and a held-out subset of Stage 1 and were then fixed for every later stage. The Stage-1 subset was used only for continual-learning model selection. In contrast, the open-world gate normalization statistics, fusion weights, and decision threshold were estimated exclusively from the FF++ source-validation calibration episodes described in Section 4.5. These quantities were frozen before target-domain evaluation and were not re-estimated using Stage 1 or any other target-domain data.

### 5.3. Baselines and Metrics

The static baselines are Xception, SBI, UCF, AltFreezing, TALL, MINTIME, VLB, and the frozen Stage-0 detector. Continual-learning baselines include naive fine-tuning, EWC, LwF, iCaRL, DER++, and adapter-only fine-tuning. All results included in the matched-protocol comparison were obtained within our experimental pipeline. We used the authors’ official implementations when available and otherwise reproduced the methods from their published specifications. Architecture-specific settings were initialized from the corresponding publications and tuned only on the common validation data. Previously published values, when mentioned for context, are explicitly marked as reported results and are not included in the matched statistical comparison.

All retrained methods use the same Stage-0 initialization where applicable, dataset partitions, stage order, labeled-video budget *K*, replay budget, frame sampling, preprocessing, augmentation pipeline, evaluation metrics, and three random seeds. Unless a method requires a setting defined by its original formulation, it is optimized for 15 epochs using AdamW with an initial learning rate of $5\times 10^{-5}$, weight decay of 0.05, a batch size of eight video clips, a five-epoch linear warm-up, cosine learning-rate decay, and class-balanced mini-batches. Hyperparameters are selected using the FF++ validation set and the held-out Stage-1 validation subset and are then fixed for all later stages. No target test sample is used for tuning, model selection, or calibration.

Naive fine-tuning updates the complete detector and classification heads using only the labeled samples from the current stage, without replay, regularization, or knowledge distillation. EWC uses the same trainable parameters and adds a quadratic penalty weighted by the diagonal Fisher information estimated from the previously deployed model, with $\lambda_{\mathrm{EWC}}=100$. LwF freezes the previous-stage detector as the teacher and combines the current-stage classification loss with output distillation using temperature T=2 and $\lambda_{\mathrm{LwF}}=1$.

For replay-based comparisons, iCaRL and DER++ use the same fixed-replay capacity of B=100 embeddings as CDD-RFLNet. iCaRL selects representatives by herding and applies equal weights to current-stage classification and old-class distillation. DER++ stores the previous logits with each replay embedding and uses replay-classification and logit-matching coefficients of 0.5 and 0.5, respectively. The memory is rebalanced uniformly across all genuine and known manipulation classes whenever a new class is introduced.

The adapter-only baseline uses the same LoRA insertion locations and ranks as CDD-RFLNet: r=8 for the RGB and temporal projections and r=4 for the residual branch. The Stage-0 backbone and earlier adapters remain frozen, and only the current-stage adapters, selected normalization parameters, and output heads are optimized. It excludes the stability–plasticity teachers, reliability-weighted distillation, disagreement supervision, and prototype replay, thereby isolating the effect of parameter-efficient adaptation.

For the open-set comparisons, maximum softmax probability, energy scoring, Mahalanobis prototype distance, and open-set supervised contrastive learning use the same FF++ source-validation partition for threshold selection and hyperparameter tuning. Their settings are frozen before evaluation on unseen datasets, and no target-domain sample is used for calibration. The principal comparison tables report mean ± standard deviation across the same three runs for every method. Thus, differences in the reported results reflect the learning strategy rather than unequal access to data or different evaluation protocols.

## 6. Experimental Results and Analysis

### 6.1. Stage-0 Detection Performance

Table 3 evaluates the detector before any continual update. The stage-0 model achieves 99.7% AUC on FF++ and an average zero-shot AUC of 88.8% across the five unseen domains. Compared with VLB, the proposed model improves cross-domain AUC by 2.6 percentage points and corrupted-video AUC by 2.2 points. The improvement is more pronounced at the strict operating point: TPR@1%FPR increases from 40.6% to 49.3%. At the same time, ECE decreases from 4.2% to 2.1%, indicating that the gain is accompanied by better probability calibration. The paired hierarchical bootstrap confirms a 2.6-point cross-domain improvement over VLB (95% CI: [1.8, 3.4], p<0.001). The stage-0 detector, therefore, provides a strong and calibrated starting point for the sequential evaluation.

### 6.2. Continual Adaptation and Knowledge Retention

Table 4 reports performance after the complete four-stage sequence. Naive fine-tuning obtains the highest current-domain AUC of 94.1%, but its final average AUC falls to 82.6% because it forgets 15.6 points on earlier domains. EWC and LwF reduce forgetting, although their final average AUC remains below 89%. Replay-based methods are more stable: iCaRL reaches 90.0% average AUC, while DER++ reaches 91.5%. Adapter-only updating further reduces forgetting to 2.8 points, but its restricted update rule limits adaptation to the new domain.

CDD-RFLNet achieves the best balance, with a final average AUC of 93.7±0.4%, forgetting of 1.0±0.2 points, and forward transfer of 3.4 points. Its average AUC exceeds DER++ by 2.2 points (95% CI: [1.5, 2.9], p<0.001), while its forgetting is 1.8 points lower than the best competing value obtained by adapter-only training (95% CI: [1.2, 2.4], p<0.001). Although the current-domain AUC is 2.2 points below unconstrained fine-tuning, the large retention gain produces a substantially stronger model over the complete set of learned domains.

The stage-wise matrix in Table 5 shows how these aggregate results arise. Before adaptation, the frozen stage-0 model obtains 91.3%, 86.7%, 90.6%, and 86.6% AUC on CDF-v2, DFDC, DFo, and DF40-FS, respectively. After each domain is introduced, the corresponding diagonal AUC increases to 94.8%, 91.7%, 94.5%, and 91.9%. The mean adaptation gain is, therefore, 4.4 points. Retention remains stable throughout the sequence: after stage 4, FF++ decreases by only 1.3 points, CDF-v2 by 1.3 points, DFDC by 0.9 points, and DFo by 0.5 points relative to their best earlier checkpoints. This pattern shows that the model learns each new domain without a progressive collapse on the earlier tasks.

### 6.3. Recognition of Unknown Manipulations

Table 6 evaluates each target domain immediately before it is used for adaptation. Maximum softmax probability performs poorly under domain shift, producing an AUROC of 74.6% and an FPR@95TPR of 62.7%. Energy and Mahalanobis scoring improve AUROC to 80.8% and 82.5%, respectively, which confirms that both logit magnitude and feature-space distance contain useful unknownness information. Their combination reaches 86.7% AUROC, but it still misses temporal cases in which only a short part of a clip is unfamiliar.

The complete three-signal gate achieves 91.4±0.6% AUROC, 90.2±0.7% AUPR, and 83.6% OSCR, while reducing FPR@95TPR to 22.8%. Relative to energy plus prototype distance, temporal disagreement improves AUROC by 4.7 points (95% CI: [3.6, 5.8], p<0.001) and OSCR by 6.5 points. The gain is largest for clips containing brief manipulated segments, supporting the use of the maximum frame disagreement together with its temporal average.

### 6.4. Low-FPR Operation and Calibration

The authenticity threshold in Table 7 is selected once on the stage-0 validation split and is not retuned on any target dataset. This is a more demanding test than selecting a separate threshold for each domain. Fine-tuning retains only 27.6% of manipulated videos at 1% FPR and has an ECE of 10.8%. LwF, DER++, and adapter-only training improve this operating point, but remain below 45% TPR.

After the full sequence, CDD-RFLNet achieves 52.6% TPR@1%FPR and an ECE of 2.7%. Compared with DER++, this is a 7.8-point TPR improvement and a 2.1-point ECE reduction. The corresponding NLL of 0.244 shows that the calibration improvement is not caused merely by shifting the decision threshold. Risk–coverage analysis gives the same conclusion: rejecting 10% of the most unfamiliar clips reduces the retained-set error from 11.1% to 6.4%, whereas DER++ reaches 8.7% error at the same 90% coverage.

### 6.5. Ablation and Mechanism Analysis

Table 8 isolates the contribution of each mechanism. Removing the residual-prototype score has little effect on final average AUC but reduces unknown AUROC by 4.1 points, showing that residual-space distance mainly supports unknown recognition. Removing the energy term causes a similar 3.3-point decrease. In contrast, the stability teacher and prototype replay primarily protect old knowledge: their removal increases forgetting from 1.0 to 5.6 and 3.7 points, respectively.

Uniform teacher averaging reduces average AUC to 91.9% because it gives equal weight to a confident specialist and an uncertain previous model. Removing the disagreement-controlled adapter gate causes the largest combined degradation: average AUC decreases by 2.8 points, forgetting increases by 2.9 points, and unknown AUROC decreases by 4.7 points. Thus, disagreement is useful not only as an unknownness measure, but also as a localized control signal for deciding where the model should change.

The diagnostic measurements support this interpretation. Before adaptation, the Spearman correlation between the unknown score and prediction error is 0.62; after the new domain is learned, it falls to 0.39 as rejected examples move toward the known-class region. Adapter activation in the highest teacher-disagreement quartile is 2.7 times that in the lowest quartile. Randomly shuffling disagreement across frames reduces final average AUC by 1.6 points, even though its clip-level mean is unchanged. The temporal position of the disagreement, therefore, carries information beyond its average magnitude.

These controlled comparisons show that the improvement does not arise from merely placing several established components in one architecture. Uniform teacher averaging reduces average AUC from 93.7% to 91.9%, removing the disagreement-controlled adapter gate reduces it to 90.9%, and temporally shuffling disagreement causes a further 1.6-point reduction. The results, therefore, support the proposed use of disagreement as the mechanism connecting unknown recognition, distillation, and localized adaptation.

### 6.6. Effect of the Annotation Budget and Stage Order

Table 9 examines the number of verified videos available at each stage. With only ten labeled videos per emerging family, the model already reaches 90.6% final average AUC and limits forgetting to 1.4 points. Increasing the budget to 25 and 50 videos raises average AUC to 92.0% and 93.0%, respectively. The gain from 50 to 100 videos is smaller, indicating that the proposed update is most valuable in the low-label regime. Unknown AUROC also improves with the budget because better prototypes and disagreement targets are available after each update.

Five additional random stage orders produce 93.3±0.5% average AUC, 1.2±0.3 points of forgetting, and 90.9±0.6% unknown AUROC. These values remain close to the primary-order results, showing that the observed advantage is not tied to one convenient ordering of the datasets.

### 6.7. Efficiency and Failure Analysis

The stage-0 detector contains 31.4M parameters, requires 82.7 GFLOPs for a 16-frame clip, and processes 37.6 clips/s. Each stage contributes 0.42M adapter parameters. After four updates, the deployed model contains 33.1M parameters and requires 86.1 GFLOPs, corresponding to increases of 5.4% and 4.1%, respectively. Throughput decreases modestly to 35.2 clips/s. The fixed-replay memory, including compressed tokens, labels, stored logits, and prototype statistics, occupies 0.38 MB. Both teachers are removed after each update, so their parameters and computation do not contribute to deployment cost.

The remaining errors are concentrated in heavily compressed clips, very small or profile faces, severe occlusion, and tracks containing long face-detection failures. Some unfamiliar genuine-capture pipelines are also rejected because their residual statistics lie far from the source prototypes. Conversely, a new generator can escape rejection when both its temporal behavior and residual statistics closely resemble a known family. These cases indicate that the gate should be used to support forensic review and targeted data acquisition rather than as independent evidentiary proof.

Taken together, these results show that CDD-RFLNet balances three deployment requirements: recognizing unfamiliar manipulations before adaptation, learning verified new domains while limiting forgetting, and maintaining practical inference and memory costs. The remaining errors under severe compression, occlusion, face-tracking failure, and previously unseen genuine-capture conditions indicate that the gate should support forensic review and targeted data acquisition rather than serve as independent evidentiary proof.

## 7. Conclusions

This paper presented CDD-RFLNet, a continual open-world framework for deepfake-video detection under evolving manipulation methods. The framework combines RGB, constrained-residual, temporal-change, and forensic-disagreement evidence to recognize known manipulations and identify unfamiliar ones. Its source-calibrated three-signal gate supports the rejection of unknown clips, while stability–plasticity distillation, prototype replay, and lightweight adapters enable controlled learning of verified attacks without extensive parameter growth.

The experiments demonstrate improvements across detection, unknown recognition, calibration, and continual retention. Before continual adaptation, the Stage-0 model achieved 88.8±0.4% mean cross-domain AUC, 49.3±0.9% TPR at 1% FPR, and 2.1±0.2% ECE. After four continual stages, CDD-RFLNet achieved 93.7±0.4% final mean AUC with only 1.0±0.2 percentage points of forgetting. The open-world gate achieved 91.4±0.5% AUROC, 90.2±0.6% AUPR, 22.8±0.7% FPR@95TPR, and 83.6±0.5% OSCR. Together, these findings indicate that the gains are not limited to aggregate AUC: the proposed method also improves strict low-FPR operation, uncertainty estimation, recognition of unfamiliar attacks, and retention of previously learned forensic knowledge.

Several limitations remain. Performance can deteriorate under severe compression, very small or profile faces, heavy occlusion, and face-detection failure. The gate may reject genuine videos produced by previously unseen capture or processing pipelines, while a new generator whose statistics closely match a known family may evade rejection. The continual procedure also assumes access to a limited number of verified labels and a finite replay memory, and each update introduces additional training and storage cost. Consequently, the gate should be used to support forensic review and targeted data acquisition rather than as independent evidentiary proof.

Future work will investigate compression-aware and face-detector-independent cues, privacy-preserving replay or prototype memory, calibration under gradual domain drift, mixed and partially labeled manipulation streams, adaptive attacks, and replay-buffer poisoning. Our next short-term goal is a fully online, drift-aware version of CDD-RFLNet that initiates adaptation only after persistent distribution change is detected and filters poisoned or low-confidence samples before they enter the review and replay buffers. This project extension will be evaluated using long, non-stationary video streams to measure detection delay, false-update frequency, computational cost, calibration drift, and long-term forgetting.

## Figures and Tables

**Table 1:** Video allocation across the continual-learning stages. FF++: FaceForensics++; CDF-v2: Celeb-DF-v2; DFDC: Deepfake Detection Challenge; DFo: DeeperForensics-1.0; and DF40-FS: the face-swapping subset of DF40. At Stages 1–4, K∈{10,25,50,100} denotes the number of labeled adaptation videos from each newly introduced manipulation family.

Stage	Dataset	Introduced Classes	Training	Validation	Test
0	FF++ c23	Genuine plus four FF++ families	3600	700	700
1	CDF-v2	Emerging Stage-1 family	*K*	100	500
2	DFDC	Emerging Stage-2 family	*K*	100	500
3	DFo	Emerging Stage-3 family	*K*	100	500
4	DF40-FS	Emerging Stage-4 family	*K*	100	500

**Table 2:** Training and implementation settings used in the matched-protocol experiments. Settings are shared by the proposed method and retrained baselines unless a method-specific value is listed.

Setting	Value
Video input	16 uniformly sampled aligned RGB frames, 224×224 pixels
Preprocessing	Face alignment, resizing, and ImageNet normalization
Training augmentation	Shared class-balanced spatial, compression, and temporal augmentation pipeline
Optimizer	AdamW
Initial learning rate	$5\times 10^{-5}$
Weight decay	0.05
Mini-batch size	8 video clips
Plasticity-teacher training	10 epochs
Continual-student/baseline training	15 epochs
Learning-rate schedule	5-epoch linear warm-up followed by cosine decay
Replay capacity	B=100 embeddings
Adapter ranks	RGB/temporal: r=8; residual: r=4
Model selection	FF++ validation set and held-out Stage-1 validation subset
Open-world calibration	FF++ source-validation calibration episodes only
Independent runs	Three random seeds

**Table 3:** Stage-0 video-level results before continual adaptation. FF++: FaceForensics++; CDF-v2: Celeb-DF-v2; DFDC: Deepfake Detection Challenge; DFo: DeeperForensics-1.0; WDF: WildDeepfake; and DF40-FS: the face-swapping subset of DF40. Cross AUC is the mean over CDF-v2, DFDC, DFo, WDF, and DF40-FS; and Corr. is the mean AUC on corrupted FF++ videos. Results are reported as mean ± standard deviation over three independent runs. Throughput is a single engineering measurement. All values except throughput are percentages. Boldface indicates the best result in each column. An downward arrow indicates that lower values are better.

Method	FF++	Cross	Corr.	TPR@1%	ECE ↓	Clips/s
MINTIME	99.6±0.1	84.3±0.6	91.6±0.4	33.9±1.2	5.1±0.3	31.8
VLB	99.6±0.1	86.2±0.5	92.6±0.4	40.6±1.0	4.2±0.2	34.2
CDD-RFLNet ($S_{0}$)	99.7±0.1	88.8±0.4	94.8±0.3	49.3±0.9	2.1±0.2	**37.6**

**Table 4:** Continual-learning results after the complete four-stage sequence. Results are reported as mean ± standard deviation over three independent runs. Average AUC and Current AUC are percentages; and BWT and FWT are measured in percentage points. Higher Average AUC, Current AUC, BWT, and FWT are better, whereas lower forgetting is better. Boldface indicates the best result in each column. An upward arrow indicates that higher values are better, whereas a downward arrow indicates that lower values are better.

Method	Avg. AUC ↑	Current AUC ↑	Forget ↓	BWT ↑	FWT ↑
Fine-tuning	82.6±0.8	94.1±0.5	15.6±0.9	−15.6±0.9	2.5±0.4
EWC	86.9±0.7	92.0±0.6	10.2±0.7	−10.2±0.7	2.0±0.3
LwF	88.4±0.6	92.4±0.5	7.9±0.6	−7.9±0.6	2.2±0.3
iCaRL	90.0±0.5	92.8±0.5	5.8±0.5	−5.8±0.5	2.5±0.3
DER++	91.5±0.5	93.1±0.4	3.4±0.4	−3.4±0.4	2.8±0.3
Adapter-only	91.2±0.5	91.7±0.5	2.8±0.4	−2.8±0.4	2.9±0.3
CDD-RFLNet	93.7±0.4	91.9±0.4	1.0±0.2	−1.0±0.2	3.4±0.3

**Table 5:** Stage-wise video AUC (%). Rows denote the checkpoint after each adaptation stage; columns denote domains already learned at that stage. FF++: FaceForensics++; CDF-v2: Celeb-DF-v2; DFDC: Deepfake Detection Challenge; DFo: DeeperForensics-1.0; and DF40-FS: the face-swapping subset of DF40.

Checkpoint	FF++	CDF-v2	DFDC	DFo	DF40-FS
$S_{0}$	99.7	–	–	–	–
$S_{1}$	99.2	94.8	–	–	–
$S_{2}$	98.9	94.2	91.7	–	–
$S_{3}$	98.6	93.8	91.1	94.5	–
$S_{4}$	98.4	93.5	90.8	94.0	91.9

**Table 6:** Unknown-manipulation recognition before adaptation, macro-averaged over stages 1–4. All values are percentages. Boldface indicates the best result in each column. An upward arrow indicates that higher values are better, whereas a downward arrow indicates that lower values are better.

Unknown Score	AUROC ↑	AUPR ↑	FPR95 ↓	OSCR ↑
Max softmax	74.6	71.8	62.7	64.9
Energy	80.8	78.4	49.6	70.8
Mahalanobis	82.5	80.1	45.9	72.4
Open-set SupCon	84.1	82.0	41.7	74.3
Energy + prototype	86.7	84.8	34.6	77.1
CDD-RFLNet gate	**91.4**	**90.2**	**22.8**	**83.6**

**Table 7:** Final-stage operational reliability. The decision threshold is fixed on the stage-0 validation set. All values except NLL are percentages. Boldface indicates the best result in each column. An upward arrow indicates that higher values are better, whereas a downward arrow indicates that lower values are better.

Method	AUC ↑	BAcc ↑	TPR@1% ↑	ECE ↓	NLL ↓
Fine-tuning	82.6	75.8	27.6	10.8	0.515
LwF	88.4	82.9	38.7	6.9	0.382
DER++	91.5	86.0	44.8	4.8	0.318
Adapter-only	91.2	85.6	43.5	4.5	0.326
CDD-RFLNet	**93.7**	**88.9**	**52.6**	**2.7**	**0.244**

**Table 8:** Component ablation under the same stage order, label budget, and fixed-replay-token budget. All values are percentages or percentage points. Boldface indicates the best result in each column. An upward arrow indicates that higher values are better, whereas a downward arrow indicates that lower values are better.

Variant	Avg. AUC ↑	Forget ↓	Unknown AUROC ↑	ECE ↓
w/o residual-prototype score	93.4	1.1	87.3	2.9
w/o energy score	93.5	1.1	88.1	3.0
w/o stability teacher	90.8	5.6	90.7	4.8
w/o prototype replay	91.6	3.7	90.9	3.9
Uniform teacher averaging	91.9	2.7	89.8	4.1
w/o disagreement adapter gate	90.9	3.9	86.7	4.6
CDD-RFLNet	**93.7**	**1.0**	**91.4**	**2.7**

**Table 9:** Sensitivity to the number *K* of labeled videos per emerging family. Results are measured after the final stage. Boldface indicates the best result in each column. An upward arrow indicates that higher values are better, whereas a downward arrow indicates that lower values are better.

*K*	Current AUC ↑	Avg. AUC ↑	Forget ↓	Unknown AUROC ↑
10	89.1	90.6	1.4	89.8
25	90.5	92.0	1.2	90.4
50	91.3	93.0	1.1	91.0
100	**91.9**	**93.7**	**1.0**	**91.4**

## Data Availability

The publicly available datasets analyzed in this study are described and cited in Section 5. The code and trained models are available from the corresponding author upon reasonable request.
